# Prenatal Exposure to Toxic Metals and Early Neurodevelopmental Outcomes in Infants Following Intrauterine Blood Transfusion: A Prospective Cohort Study

**DOI:** 10.3390/toxics13121055

**Published:** 2025-12-05

**Authors:** Iman Al-Saleh, Hissah Alnuwaysir, Mais Gheith, Reem Al-Rouqi, Hesham Aldhalaan, Eiman Alismail, Maha Tulbah

**Affiliations:** 1 Environmental Health Program, King Faisal Specialist Hospital and Research Centre, P.O. Box 3354, Riyadh 11211, Saudi Arabia; 2 School of Medicine, Alfaisal University, P.O. Box 50927, Riyadh 11533, Saudi Arabia; 3 Center for Autism Research, Obstetrics & Gynecology Department, King Faisal Specialist Hospital and Research Centre, P.O. Box 3354, Riyadh 11211, Saudi Arabia; 4 Maternal-Fetal Medicine, Obstetrics & Gynecology Department, King Faisal Specialist Hospital and Research Centre, P.O. Box 3354, Riyadh 11211, Saudi Arabia

**Keywords:** mercury, lead, cadmium, arsenic, neurodevelopment, intrauterine blood transfusion, fetal exposure

## Abstract

Fetal exposure to toxic metals is a major public health concern, yet the contribution of intrauterine blood transfusion (IUBT) to this exposure remains unclear. This prospective cohort study assessed mercury, cadmium, lead, and arsenic levels in maternal blood, cord blood, and residual IUBT red blood cell (RBC) units from 90 pregnant women enrolled at King Faisal Specialist Hospital & Research Centre. Metals were detected in nearly all maternal and cord blood samples and in every transfusion bag, with several measurements exceeding established benchmark values. Higher maternal mercury and combined mercury–arsenic levels were suggestively associated with small reductions in personal–social scores (approximately −3% to −5%). Elevated cord mercury, arsenic, and combined mercury–arsenic–cadmium levels were associated with modest decreases in problem-solving performance. Increased mercury and mercury–arsenic concentrations in transfused RBCs were linked to lower gross motor scores. Overall, these patterns indicate a potential contribution of IUBT-related metals to fetal exposure, although effect sizes were small. These preliminary findings highlight the importance of monitoring metal content in transfusion materials and reinforce the need for larger studies to clarify their clinical relevance.

## 1. Introduction

Exposure to toxic chemicals during early development is implicated in the etiology of various neurological disorders—including learning disabilities, dyslexia, intellectual disability, attention-deficit/hyperactivity disorder, and autism—arising either directly from toxicity or via gene–environment interaction [1,2,3,4]. The National Research Council estimates that 3% of neurobehavioral disorders are directly caused by environmental toxicants, with an additional 25% involving gene–environment interplay [5]. In Saudi Arabia, epidemiological data on developmental disabilities remain limited: Al-Hazmy et al. [6] reported a 1.8% prevalence of learning disabilities, while Al Salloum et al. [7] found that 0.69% of children had major neurological disorders, 0.26% had intellectual disability, and 0.23% had cerebral palsy. Consanguinity contributes to disability risk [8], but the role of environmental toxicants—particularly mercury, arsenic, cadmium, and lead—is poorly characterized.

The four target metals are well-established neurotoxicants owing to their persistence and bioaccumulation, with toxic effects primarily mediated by oxidative stress and multi-organ injury [9,10,11,12,13]. Arsenic and cadmium are classified as Group 1 carcinogens and have been associated with nephrotoxicity, placental telomere shortening, and impaired neurobehavioral performance in infants [14,15,16,17,18,19]. Lead exposure remains a primary global health concern; even low concentrations adversely affect cognitive and behavioral development and increase the risk of adverse birth outcomes [20,21,22,23,24]. Mercury exposure occurs through dietary, cosmetic, and medical pathways, and all major forms are recognized to pose risk for neurodevelopment and renal toxicity [25,26,27,28,29,30].

Intrauterine blood transfusion (IUBT) is a critical treatment for severe fetal anemia, with survival rates exceeding 90% [31,32]; however, its long-term neurodevelopmental implications remain poorly understood [33,34,35]. Although transfused blood undergoes infectious disease screening [36], it is not routinely evaluated for toxic metals, despite evidence that fetuses, neonates, and preterm infants are highly susceptible to metal contamination in blood products [37,38,39]. Lead contamination in RBC units may exceed recommended thresholds: Gehrie et al. [40] documented concentrations exceeding pediatric safety limits by more than 20-fold. The German Human Biomonitoring Commission (HBM Commission) previously established a blood lead reference level of 7 µg/dL [41,42,43], whereas the current Centers for Disease Control and Prevention (CDC) pediatric reference value is 3 µg/dL, though no level is considered safe [44,45]. Despite these concerns, the extent of transfusion-related metal exposure remains inadequately investigated.

To address this gap, we conducted a prospective observational cohort study of pregnant women receiving care at King Faisal Specialist Hospital & Research Centre (KFSH&RC). Our primary objective was to examine whether prenatal exposure to the four target metals is associated with early neurodevelopment in infants and to determine whether IUBT represents an additional pathway of fetal exposure. These metals were selected based on their well-documented developmental toxicity, placental transfer, and prior evidence of contamination in maternal–fetal biomatrices and blood products. A subgroup undergoing IUBT provided an opportunity to assess whether transfusion represents an additional—and understudied—source of fetal exposure. Building on our prior demonstration of elevated lead concentrations in transfusion units from this cohort [46], we hypothesized that higher prenatal concentrations of these metals would be associated with poorer neurodevelopmental outcomes and that IUBT may contribute additional exposure relevant to fetal risk.

## 2. Materials and Methods

### 2.1. Study Design

This prospective observational cohort study enrolled 90 pregnant women attending the Obstetrics and Gynecology Clinic at KFSH&RC between June 2020 and 2023. All eligible participants were recruited during routine third-trimester antenatal visits (weeks 32–34) and followed through to delivery. During the study period, some pregnancies required IUBT for the treatment of fetal anemia at the maternal–fetal therapy unit. These women formed a naturally occurring subgroup within the cohort, allowing assessment of IUBT as a potential additional source of fetal metal exposure.

Venous blood samples (maternal) were collected during the third trimester, and cord blood samples were obtained immediately after delivery. For pregnancies involving IUBT, residual RBCs from the transfusion units were also collected to quantify metal concentrations. The volume of blood transfused during each IUBT was individualized according to fetal weight and pretransfusion hemoglobin levels.

In total, 29 maternal venous samples, 21 cord blood samples, and residual RBCs from 29 transfusion units were analyzed. Participants received between one and nine IUBTs during pregnancy. Complete residual RBC samples were available for 18 participants; one or two units were missed for 11 cases due to logistical factors beyond the investigator’s control.

The inclusion criteria comprised age ≥ 18 years, singleton pregnancy, planned delivery at KFSH&RC, and residence in Riyadh for at least 1 year. Both parents provided written informed consent. Exclusion criteria included major chronic diseases (hypertension, diabetes, autoimmune, cardiac, or renal disorders) and pregnancy complications such as preeclampsia that developed during follow-up.

All procedures and analyses were conducted in accordance with institutional and international ethical standards and approved by the KFSH&RC Research Ethics Committee (RAC #2200007).

### 2.2. Blood Sample Collection

Venous blood samples (5 mL) were collected from each participant in ethylenediaminetetraacetic acid tubes (Becton Dickinson, Franklin Lakes, NJ, USA) during the third trimester (32–34 weeks of gestation). Cord blood (4 mL) was obtained in identical tubes immediately after delivery. From each whole-blood sample, a 200-µL aliquot was transferred into 1.5-mL cryogenic vials (Corning Incorporated, NY, USA) and stored at −30 °C until analysis for lead, cadmium, mercury, and arsenic.

For pregnancies requiring IUBT, residual blood from the transfused RBC units was also collected after each procedure to quantify metal concentrations. The volume of blood administered per IUBT was individualized based on estimated fetal weight and pretransfusion hemoglobin levels. All samples were handled using trace-metal–free materials to minimize contamination.

### 2.3. Blood/RBC Sample Preparation and Analysis

Details of sample preparation, instrumentation, calibration, accuracy checks, and analytical precision are summarized in Table 1. Briefly, whole blood and residual RBC samples were diluted 1:50 in a nitric acid–based matrix and analyzed for the four target metals using inductively coupled plasma–mass spectrometry (ICP-MS; NexION^®^ 2000, PerkinElmer, Shelton, CT, USA) with appropriate internal standards and certified reference materials. Calibration, recovery, and precision procedures followed established trace-metal quality-assurance protocols. Limits of Detection (LOD) were calculated as the standard deviation of ten blank replicates multiplied by Student’s t-value at 99% confidence. Limits of Quantification (LOQ) were calculated as 10× the standard deviation (SD) of blank replicates.

### 2.4. Two-Month Follow-Up

At a corrected age of two months (median: 4.2 months), parents of all 90 infants were invited to complete the Ages and Stages Questionnaire, Third Edition (ASQ-3), to screen for early neurodevelopmental delays. Age correction for prematurity was performed using the “Add to or subtract from a date” calculator (timeanddate.com). All ASQ-3 domain scores—including the personal–social domain—were calculated using corrected age for infants born before 37 weeks’ gestation, in accordance with ASQ-3 scoring guidelines. The ASQ-3 comprises 30 items across five developmental domains—communication, gross motor, fine motor, problem-solving, and personal–social—each rated on a three-point scale (“Yes” = 10, “Sometimes” = 5, “Not yet” = 0). Parents completed the questionnaire in approximately 10 to 15 min. Of the 90 participants, 73 (81%) returned completed ASQ-3 forms.

### 2.5. Statistical Analysis

Data were analyzed within a prospective cohort framework, with IUBT treated as a clinical exposure variable rather than a sampling criterion. Descriptive statistics were used to summarize participants’ sociodemographic and clinical characteristics, as well as metal concentrations in maternal blood, cord blood, and residual RBC transfusions. Continuous variables are presented as medians (with minimum and maximum values), and categorical variables are presented as frequencies and percentages. Comparisons between women who underwent IUBT and those who did not were performed to describe, but not define, exposure subgroups.

Values below the LOD were replaced with one-half of the LOD. Metal concentrations and ASQ-3 domain scores were log-transformed to improve normality. Group differences were assessed using the Mann–Whitney U test for continuous variables and the chi-squared test for categorical variables.

Spearman’s rank correlation coefficients were computed to evaluate associations between metal concentrations across matrices (maternal, cord, and transfusion blood) and ASQ-3 domain scores.

Principal component analysis (PCA) was performed separately for metals measured in maternal blood, cord blood, and transfusion samples to identify correlated exposure patterns. Sampling adequacy was verified using the Kaiser–Meyer–Olkin (KMO) test (KMO > 0.5), and Bartlett’s test of sphericity was applied to confirm matrix suitability. Components with eigenvalues > 1 were retained, and loadings ≥ ±0.50 were used to identify dominant metals in each component.

Multivariable linear regression models were fitted to estimate associations between log-transformed metal concentrations (and PCA components) and ASQ-3 domain scores. Analyses were conducted in two steps: first, without adjustment for IUBT to assess baseline associations; and second, with IUBT included as a covariate to evaluate how controlling for transfusion exposure affected the relationships between metal exposure levels and neurodevelopmental outcomes. All models were adjusted for potential confounders, including maternal age, body mass index (BMI), gestational age at delivery, family income, and maternal health status. For models evaluating metals measured in RBC transfusion samples, the number of RBC units received during IUBT was additionally included as a covariate to account for cumulative transfusion-related exposure. Regression coefficients were back-transformed and expressed as percentage changes (Δ%) in each ASQ-3 score per unit increase in the log-transformed metal concentration using the formula: Δ% = [exp(β) − 1] × 100.

Given the exploratory nature of the study, no formal correction for multiple comparisons was applied. Because strict methods such as Bonferroni or false discovery rate (FDR) can substantially increase the risk of type II error in studies with correlated exposures, results were interpreted using effect sizes, confidence intervals, and consistency across matrices, rather than *p*-values alone [47,48].

Given the modest and unbalanced sample size, particularly for infants with complete ASQ-3 data (*n* = 73) and those who underwent IUBT (*n* = 29), the study was likely underpowered to detect small effect sizes. Because post hoc power calculations for multivariable models with continuous exposures may be misleading, effect sizes and confidence intervals are emphasized, and the potential for type II error is acknowledged.

All analyses were performed using SPSS Statistics for Windows, version 25.0 (IBM Corp., Armonk, NY, USA), and two-sided *p*-values < 0.05 were considered statistically significant.

## 3. Results

### 3.1. Characteristics and Exposure Profile of the Study Population

Table 2a presents the characteristics of the participants, who were categorized into two groups based on their receipt of IUBTs. Compared with those who did not receive transfusions, women who received IUBTs tended to be older and had a higher rate of live births; however, they had infants with lower gestational ages at birth and smaller anthropometric measurements (birth weight, birth height, head circumference, and crown–heel length). Furthermore, they scored lower on the 1- and 5-min Apgar tests. The number of participants in most categorical variables was limited to specific cells. Significant differences were observed between the groups regarding work status and educational levels, with a higher proportion of women who did not receive IUBTs holding university degrees and being currently employed.

As shown in Table 2b, no significant differences in metal levels were observed between the groups, except for cadmium in cord blood, which was significantly higher among the IUBT recipients (*p* = 0.003). A total of 30 women, aged 30–46 years, underwent 1–9 IUBTs per fetus for the treatment of fetal anemia caused by Rh isoimmunization. The transfusion volume for each procedure ranged from 8 to 80 mL and was determined by fetal weight, gestational age, pre-transfusion hemoglobin level, rate of hemoglobin decline, and the presence of hydrops fetalis. IUBTs were performed between 18 and 33 weeks of gestation. The median pre-, mid-, and post-transfusion hemoglobin levels (g/dL) were 6.2 (1.2–14), 11.6 (2.8–17.7), and 14.3 (7.1–18.2), respectively.

We analyzed 124 RBC transfusion units, confirming that metals were detected and quantified. Detection frequencies were high: 98% for mercury, 90% for cadmium, 100% for lead, and 90% for arsenic. The median concentrations (range) of the four target metals in transfused RBC units were: 0.746 (0.001–28.225) for mercury, 0.749 (0.001–3.947) for cadmium, 12.798 (3.116–41.439) for lead, and 0.436 (0.0007–8.423) µg/L for arsenic. These results confirm that transfusion units contained quantifiable levels of all four metals, reinforcing transfusion products as a potential contributor to fetal exposure.

### 3.2. ASQ-3 Assessment and Relationship with Risk Factors

**Figure 1:**
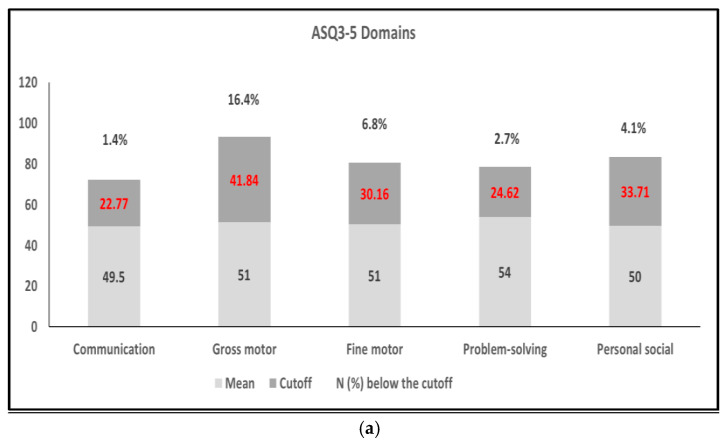

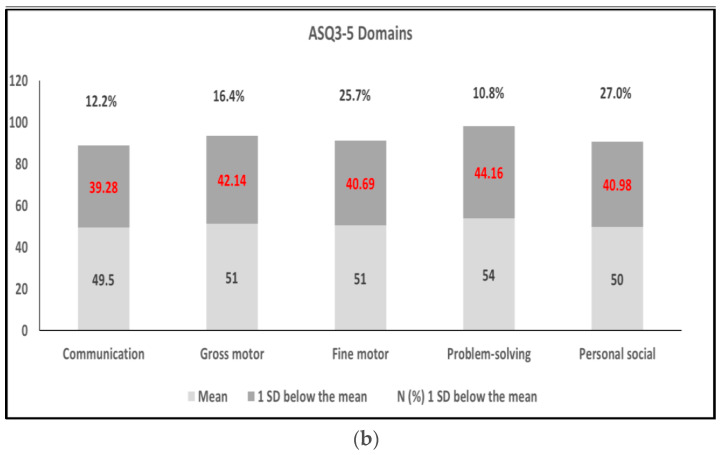
Neurodevelopmental assessment using the Ages & Stages Questionnaire, Third Edition (ASQ-3), at a corrected age of 2 months (*n* = 30). (**a**) displays the distribution of infant scores across the five ASQ-3 developmental domains (communication, gross motor, fine motor, problem-solving, and personal–social) relative to established ASQ-3 cutoff values. (**b**) shows the proportion of infants scoring one standard deviation below the mean, indicating areas of potential developmental concern. All domain labels and thresholds are highlighted in red for clarity.

As shown in Figure 1, among the 74 infants evaluated, the number with scores below the cut-off values were as follows: 1 in communication (cut-off = 22.77), 12 in gross motor (cut-off = 41.84), 5 in fine motor (cut-off = 30.16), 2 in problem-solving (cut-off = 24.62), and 3 in personal–social (cut-off = 33.71). A total of 17 infants (~23%) had low scores in one domain (*n* = 10) or two domains (*n* = 7). A score below 1SD was observed in the following ASQ-3 domains: communication (9 infants), gross motor (12 infants), fine motor (19 infants), problem-solving (8 infants), and personal–social (6 infants). As shown in Table 2a, there were no statistically significant differences in the five ASQ-3 domains of infants with and without receipt of IUBT. For scores below these cut-off values, the ratios of infants who received IUBT to those who did not were 0:1, 4:8, 2:3, 1:1, and 1:2 for communication, gross motor, fine motor, problem-solving, and personal–social, respectively. Twelve of the infants with low scores did not receive IUBT.

### 3.3. Risk Factors Associated with the Five Domains of the ASQ-3 and/or Metal Exposure

Spearman’s rank correlation analysis revealed statistically significant but modest negative associations between the following variables: problem-solving scores and mercury levels in maternal blood (rs = −0.269, *p* = 0.02, *n* = 74), communication scores and cadmium levels in cord blood (rs = −0.426, *p* = 0.003, *n* = 47), and problem-solving scores and arsenic levels in cord blood (rs = −0.495, *p* < 0.001, *n* = 47). Conversely, suggestive positive correlations were found between problem-solving scores and lead levels in maternal blood (rs = 0.255, *p* = 0.028, *n* = 74) and between fine motor scores and lead levels in cord blood (rs = 0.305, *p* = 0.037, *n* = 47). We investigated ASQ-3 scores in 18 infants and the levels of metals in their transfusions. We observed inverse associations between the communication scores and RBC mercury (rs = −0.529, *p* = 0.024) and arsenic (rs = −0.482, *p* = 0.043). Similar inverse relationships were observed between the problem-solving scores and levels of mercury (r = −0.519, *p* = 0.027) and arsenic (r = −0.485, *p* = 0.041).

Positive correlations were observed between the levels of mercury, lead, and arsenic in the maternal blood and their counterparts in the cord blood. Spearman’s rank correlation coefficients were as follows: rs = 0.541 (*p* < 0.001) for mercury; rs = 0.318 (*p* = 0.03) for lead; and rs = 0.566 (*p* < 0.001) for arsenic.

In cord blood, cadmium levels were positively associated with maternal age (rs = 0.309, *p* = 0.035, *n* = 47) but negatively associated with gestational age at birth (rs = −0.361, *p* = 0.013, *n* = 47) and head circumference (rs = −0.533, *p* = 0.033, *n* = 47). Meanwhile, mercury was negatively associated with maternal body mass index (rs = −0.321, *p* = 0.028, *n* = 47). In maternal blood, mercury levels were negatively correlated with newborn weight (r = −0.246, *p* = 0.049, *n* = 65), whereas arsenic levels were negatively associated with gestational age at birth (r = −0.246, *p* = 0.035, *n* = 74). The results, including the general characteristics, the ASQ-3 five domains, and heavy metals in maternal and cord blood, are shown in Table 2a,b.

### 3.4. Principal Component Analysis (PCA)

The correlations of the four metals in maternal blood were further examined via PCA to assess pregnant women’s exposure profile during the third trimester. Two PCs were extracted, dominated by mercury and arsenic, accounting for 33.33% of the total variance, and PC2, characterized by lead and cadmium, representing 29.137%. The KMO was 0.437, and Bartlett’s test statistic was 12.626 (*p* < 0.05), indicating adequate sample adequacy for PCA.

Similarly, we analyzed cord blood to evaluate metals for fetal exposure. In this analysis, PC1, comprising mercury, arsenic, and cadmium, accounted for 43.528% of the variance, whereas PC2, consisting solely of lead, accounted for 26.012%. The KMO was 0.596, and Bartlett’s test statistic was 19.591 (*p* < 0.01), supporting the appropriateness of PCA for these data.

The PCA results were also significant for metals in RBC transfusions, indicating exogenous exposure related to the IUBT procedure. PC1, which included mercury and arsenic, accounted for 35.963% of the total variance, whereas PC2, consisting of lead and cadmium, accounted for 26.678%. The KMO was 0.526, and Bartlett’s test statistic was 38.313 (*p* < 0.001), indicating that PCA was suitable for these data.

**Figure 2:**
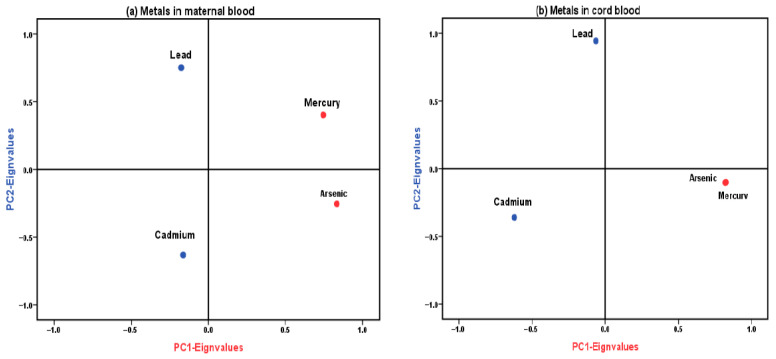

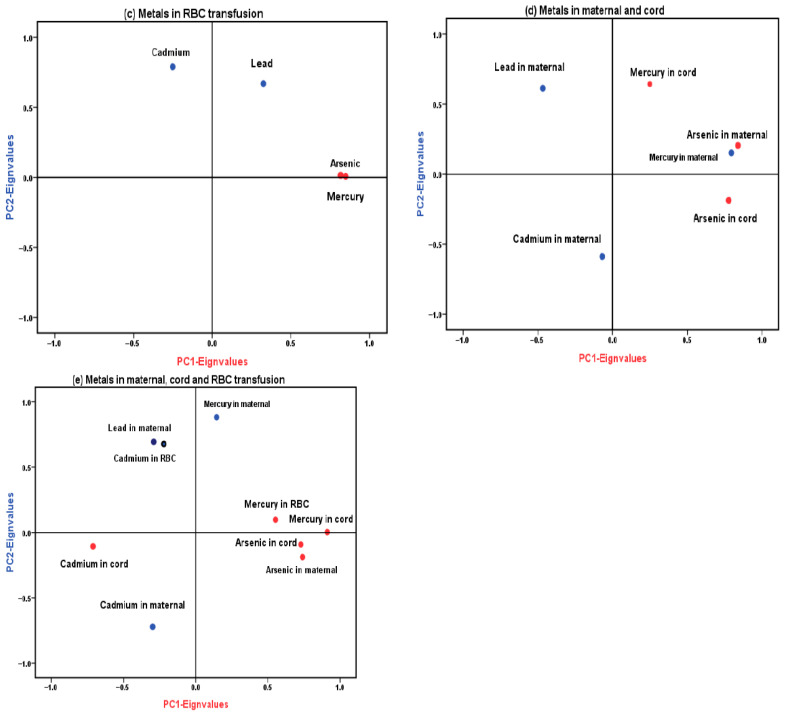
Rotated principal component loading plots illustrating metal co-exposure patterns based on principal component analysis (PCA). Panels depict loading vectors for metals measured in (**a**) maternal blood, (**b**) cord blood, (**c**) combined maternal + cord blood datasets, (**d**) intrauterine transfused red blood cells (RBCs), and (**e**) the combined dataset of maternal, cord, and RBC metals. Red vectors represent Principal Component 1 (PC1), and blue vectors represent Principal Component 2 (PC2). Only metals with factor loadings ≥ 0.50 are displayed, indicating strong contributions to the corresponding component. These loading patterns reflect exploratory co-exposure structures rather than definitive latent components.

In addition, we combined metals in the cord and maternal blood as an indicator of prenatal exposure, which showed a KMO of 0.658 and Bartlett’s test statistic of 76.230 (*p* < 0.001), with PC1 comprising arsenic in the maternal, mercury in the cord, arsenic in the cord, and cadmium in the cord blood. Meanwhile, PC2 accounted for 17.544% of mercury, lead, and cadmium in the maternal blood. We analyzed metals in three matrices—maternal blood, cord blood, and RBC transfusion—and obtained a KMO measure of 0.390 and Bartlett’s test statistic of 101.448 (*p* = 0.003). In this analysis, PC1, which included mercury and arsenic, accounted for 27.804% of the total variance. Contrarily, PC2, which consisted of mercury, lead, and cadmium from maternal sources and RBC transfusion, accounted for 19.943%. These results are presented in Figure 2.

### 3.5. Regression Models

We used separate linear regression models to assess the relationships between ASQ-3 domain scores, various factors, and metal exposure with and without adjustment for IUBT. Our analysis considered the impact of individual and combined metals measured in maternal blood, cord blood, and RBC transfusions, adjusting for confounders and risk factors.

#### 3.5.1. Initial Analysis Without the IUBT

The increased mercury levels in maternal blood were associated with modest reductions in the ASQ-3 personal–social scores (β = −0.031, 95% CI = −0.056 to −0.007, *p* = 0.013). Elevated arsenic in cord blood was also correlated with lower problem-solving scores (β = −0.04, 95% CI = −0.068 to −0.012, *p* = 0.006). In PCA of combined metals, PC1 (mercury–cadmium–arsenic) showed a statistically significant but modest association with decreased problem-solving scores (β = −0.089, 95% CI = −0.172 to −0.006, *p* = 0.037). Conversely, PC2 (lead–cadmium) in maternal blood was positively associated with increased problem-solving scores (β = 0.069, 95% CI = 0.008 to 0.129, *p* = 0.028). Borderline negative relationships were observed between the problem-solving scores and combinations of cadmium–lead in maternal blood, and between the personal–social scores and the mercury–arsenic combination. The results are shown in Table 3.

#### 3.5.2. RBC Transfusion Analysis

Analysis of RBC transfusion metals showed that higher mercury was associated with modest declines in gross motor scores (β = −0.116, 95% CI = −0.22 to −0.012, *p* = 0.032), as was PC1 (mercury–arsenic) (β = −0.138, 95% CI = −0.269 to −0.007, *p* = 0.041). Marginal (borderline) negative associations were observed between gross motor scores and arsenic in RBC transfusions and between problem-solving scores and mercury. A positive association was observed between fine motor scores and RBC lead.

#### 3.5.3. Analysis Including the IUBT

Incorporating IUBT into the models resulted in stronger associations, although still modest in magnitude (Table 4). Higher maternal mercury was associated with statistically significant—but small—reductions in personal–social scores (β = −0.035, 95% CI = −0.060 to −0.010, *p* = 0.006). Similarly, statistically significant—but modest—declines in problem-solving scores were associated with elevated mercury levels in cord blood and receipt of IUBT (β = −0.063 and β = −0.364, respectively). Modest reductions were also associated with arsenic levels in the cord blood and IUBT receipt (β = −0.041 and β = −0.326, respectively) as well as with PC1 in the cord blood and IUBT receipt (β = −0.112 and β = −0.398, respectively). A similar pattern of modest declines was observed in association with PC1, representing the combination of metals in both cord and maternal blood and IUBT receipt (β = −0.101 and β = −0.390, respectively). Marginal (borderline) negative associations were also observed between communication scores and cadmium levels in the cord blood. Conversely, the problem-solving scores in maternal models were inversely associated with cadmium levels but positively associated with lead levels. Moreover, including PC1 in maternal blood showed a modest inverse association with personal–social scores for the combined mercury–arsenic exposure (β = −0.055, 95% CI = −0.101 to −0.009, *p* = 0.020).

## 4. Discussion

### 4.1. Key Findings

This prospective study investigated exposure to the four target metals in 90 pregnant women attending the Obstetric and Gynecology Clinic at KFSH&RC, of whom 29 were receiving IUBT, and evaluated the risk of neurodevelopmental delays in their infants at 2 months of age. The extent of exposure was notable: mercury was detected in 92% and 98% of maternal and cord blood samples, lead in 98% and 100%, cadmium in 97% and 70%, and arsenic in almost 100% and 64%, respectively. Furthermore, significant concentrations of heavy metals were detected in the RBC transfusion units used for IUBT. Although most levels did not exceed established safety thresholds, their presence—given the known neurotoxicity of these metals—raises concern. Interestingly, among the studied metals, only cadmium levels differed between groups, with higher cord concentrations in IUBT recipients. No group differences were observed across the five ASQ-3 domains.

Specific modest decreases in ASQ-3 scores were associated with higher concentrations of selected metals, including personal–social (−3.05%) and problem-solving (−3.9% and −8.5%). After adjusting for IUBT, these estimates remained similar, and some associations strengthened numerically. Because effect sizes were small and confidence intervals were wide in several cases, these associations should be interpreted with caution.

Associations between metal concentrations in RBC transfusions and ASQ-3 scores were also observed, including modest declines in gross motor performance associated with mercury and combined mercury–arsenic levels. These findings suggest the possibility that transfusion-derived metals may contribute to overall fetal exposure, although causal conclusions cannot be drawn.

### 4.2. The Extent of Exposure to Heavy Metals in All Pregnant Women

We obtained notable findings on exposure to heavy metals among study participants—those who received IUBT and those who did not. Specifically, mercury was detected in 92% of maternal and 98% of cord blood samples, with mean concentrations of 0.455 µg/L (median = 0.351 µg/L; min–max = 0.001–2.151 µg/L) and 0.896 µg/L (median = 0.779 µg/L; min–max = 0.001–4.72 µg/L), respectively. Although the mercury levels of the participants remained below the United States Environmental Protection Agency’s (US EPA) safe reference level of 5.8 µg/L, as established in 2007, concerns arose given the fetal safety benchmarks [49]. Some researchers [50,51] recommend a more stringent benchmark dose of 3.5 µg/L to avert the neurotoxic effects in fetuses. Notably, while the levels in all maternal samples remained below this benchmark, those in two cord blood samples exceeded it, highlighting the potential risks. In addition, a significant correlation (r = 0.57, *p* < 0.001) was observed between the mercury levels in the maternal and cord blood samples, with the levels in the latter being more than twice as high as those in the former—likely due to the high-affinity fetal-specific serum albumin proteins present in cord blood [52,53,54,55,56]. Interestingly, the current median mercury levels are lower than those reported over a decade ago, which were 1.949 and 2.876 µg/L for cord and maternal blood, respectively, indicating a potential decrease in overall exposure levels within the studied population [52]. Despite this reduction, the in utero exposure to mercury remains a concern, mainly as the mercury levels in our study were substantially higher than the median reported by the National Health and Nutrition Examination Survey (NHANES), which was below the minimum detection limit of 0.28 µg/L for children aged 1 to 5 years [57].

Lead exposure was similarly prevalent, detected in 98% of maternal and 100% of cord blood samples, with mean concentrations of 5.713 µg/L (median = 4.236 µg/L; min–max = 0.022–82.418) and 4.015 µg/L (median = 3.254 µg/L; min–max = 0.233–12.053 µg/L), respectively. Remarkably, the median lead concentration in maternal samples was significantly higher than that in cord blood (*p* = 0.025), with a correlation of 0.425 (*p* < 0.001), confirming placental transfer and aligning with our previous findings [52]. However, only one maternal sample surpassed the CDC’s updated reference value of 35 µg/L for children [58]. Nonetheless, 33.7% of the maternal and 27.9% of the cord blood samples exceeded the more stringent interim reference level of 5 µg/L for blood lead, highlighting the critical health concerns as no level of lead exposure is considered safe [59]. Compared with NHANES (2017–2018) data, the maternal and cord blood lead levels in our study were approximately half of the NHANES median levels for children aged 1 to 5 years, which were 6.4 and 6.2 µg/L, respectively [57]. Despite our values being lower than those reported in previous Al-Kharj studies—18.2 and 20.06 µg/L—our findings are still concerning owing to the recognized neurotoxicity of lead and its classification as a probable human carcinogen, which led the German Federal Agency Commission to suspend the reference level for blood lead [43].

Cadmium was detected in 97% of the maternal and 70% of the cord blood samples, with mean concentrations of 0.638 µg/L (median = 0.19 µg/L; min–max = 0.001–4.822 µg/L) and 0.139 µg/L (median = 0.029 µg/L; min–max = 0.001–2.371 µg/L), respectively. None of the samples had cadmium levels above the 5-µg/L threshold set by the American Occupational Safety and Health Administration (OSHA) for occupational exposure [60]. However, 31.5% of maternal and 8.2% of cord blood samples exceeded the 95th percentile reference value of 0.3 µg/L, indicating potential health risks [41]. In the NHANES survey (2017–2018), cadmium blood levels were below the LOD of 0.1 µg/L in most children aged 1–5 years [57]. Our maternal and cord cadmium levels were 81% and 18%, respectively, 0.1 µg/L. Notably, the cadmium levels in the maternal blood samples were approximately seven times higher than those in the cord blood samples, suggesting that the placenta may have obstructed its transfer [52,61]. Although the median cadmium levels in our study were significantly lower than those previously reported in Saudi Arabia (0.704 and 0.983 µg/L) [52], 18% of the maternal and 5% of the cord samples had cadmium levels exceeding 1 µg/L, which was likely associated with tobacco exposure, including cases of two women who smoked and 28 who were exposed to second-hand smoke [62]. Given the long half-life (10–30 years) of cadmium and its tendency to accumulate in tissues, there is an increased potential for long-term health risks, including cancer [63].

Arsenic was detected in 99% of the maternal samples and 64% of the cord samples, with mean concentrations of 0.411 µg/L (median = 0.330 µg/L; min–max = 0.0007–1.859 µg/L) and 0.254 µg/L (median = 0.076 µg/L; min–max = 0.0007–3.558 µg/L), respectively. The arsenic levels in the maternal samples were significantly more than four times higher than those in the cord samples, with a positive correlation of 0.489 (*p* < 0.001) between the two, consistent with previous studies indicating placental transfer of arsenic from the mother to the fetus [64,65]. Despite our study’s generally low arsenic levels, seven maternal and three cord samples exceeded the Canadian Health Measures Survey reference value of 1.4 µg/L for blood arsenic for children aged 6–19 years [66]. This reference was the only one available in the literature. Moreover, the cord arsenic levels in our study were lower than those reported in other countries, such as Japan (3.65 µg/L), Jamaica (0.6 µg/L), Brazil (10.7 µg/L), China (1.53 µg/L), and South Africa (0.72 µg/L) [67,68,69,70,71]. However, it is noteworthy that our measurements were of total arsenic, not accounting for the more toxic inorganic forms or urinary methylated metabolites, which may have significantly different health implications [72].

Our study demonstrates that pregnant women are exposed to high levels of heavy metals, with detected concentrations in the maternal and cord blood samples that may pose health risks to fetal development.

### 4.3. Exposure Levels in the Context of the IUBT

Given that 29 women in our study underwent multiple IUBTs during pregnancy, we examined the possibility of increased exposure to heavy metals, a concern highlighted in the previous section. Notably, the cadmium levels were found to be approximately four times higher in recipients’ cord blood than in nonrecipients. Contrarily, other maternal and cord blood metal levels showed no significant variations. This finding is particularly unexpected given the extensive metal contamination we detected in the 124 analyzed RBC transfusion units, with several exceeding the established safety thresholds. Specifically, three units had mercury levels above the US EPA’s reference value of 5.8 µg/L [49], and eight exceeded 3.5 µg/L [51]. Although the cadmium levels did not exceed OSHA’s threshold of 5 µg/L [60] units had levels exceeding 1 µg/L, commonly found in smokers [63]. For lead, four units had concentrations over 35 µg/L [73], 89 units exceeded the pediatric safety threshold of 10 µg/L for donor RBC units [40], and 122 units exceeded the interim reference level of 5 µg/L [59]. For arsenic, 15 units exceeded the 1.4 µg/L threshold for children, and 7 surpassed the 2 µg/L threshold for adults [66].

Despite notable metal concentrations in the RBC transfusion units, the lack of differences in metal exposure between pregnant women who received and did not receive IUBT may be explained by several factors. The number and volume of transfusions could have diluted the metal concentrations in the recipients; for example, 14 fetuses received IUBT five or more times, with volumes ranging from 8 to 65 mL. The timing of blood sampling relative to transfusions can also influence detectable levels. Additionally, physiological factors such as placental transfer kinetics, rapid fetal uptake, or redistribution of metals may further obscure differences measured at delivery. Nonetheless, contaminated blood transfusions indicate a potential additional source of metal exposure for fetuses receiving IUBT. However, the extent to which this contributes to overall fetal burden remains uncertain and should be interpreted cautiously. This finding highlights the importance of monitoring and managing metal contamination in medical supplies.

Although our results raise essential concerns regarding the implications of metal exposure through transfusions, particularly in vulnerable populations, such as pregnant women and their fetuses, we did not observe measurable differences in ASQ-3 scores between infants whose mothers did or did not receive IUBT. This suggests that, within the limits of this small sample and early screening tool, the contribution of IUBT-related exposure to early developmental outcomes remains unclear.

### 4.4. Exposure and Neurodevelopment

The impact of prenatal exposure to environmental toxins, particularly heavy metals such as mercury, cadmium, arsenic, and lead, on neurodevelopment remains a critical research area. These metals have potentially deleterious effects on fetal development, influencing cognitive and behavioral outcomes in children. While the adverse effects of such exposure are recognized, findings across studies have exhibited considerable variability. This inconsistency could be attributed to differences in the study populations, methodologies, and exposure assessment techniques [3,74,75,76].

In our study, we used regression models to evaluate the impact of metal exposure without initially classifying women by receipt of IUBT. Notably, we observed modest decreases in personal–social scores of −3.05% (95% CI: −5.45% to −0.7%) associated with elevated maternal mercury levels, problem-solving scores of −3.9% (95% CI: −6.6% to −1.2%) associated with higher cord arsenic levels, and problem-solving scores of −8.5% (95% CI: −15.8% to 0−0.6%) associated with the combination of mercury, arsenic, and cadmium in cord blood samples. While these relationships remained significant after adjustment for IUBT as a binary variable, additional significant associations emerged, including modest reductions in personal–social scores of −5.4% (95% CI: −9.6% to −0.9%) and exposure to a combination of maternal mercury and arsenic, −6.1% (95% CI: −11.8% to 0%) and −9.6% (95% CI: −16.6% to −1.9%) in problem-solving scores related to cord mercury and the combination of maternal (arsenic) and cord blood (mercury, cadmium, and arsenic), respectively. Given the small effect sizes and wide confidence intervals in several models, these associations should be interpreted cautiously.

Although our results indicated that exposure to metals, particularly mercury and arsenic, was associated with modest reductions in certain ASQ-3 domains irrespective of IUBT, additional suggestive associations were observed when testing metal levels in RBC transfusion samples. A decrease in gross motor scores of 11% (95% CI: −19.8% to −1.2%) was associated with mercury, and a further 12.9% decrease (95% CI: −23.6% to −0.7%) was associated with a combination of mercury and arsenic. These findings may reflect increased vulnerability of the gross motor domain or differences in exposure matrices, though alternative explanations—including chance findings in small samples—cannot be excluded. Notably, the concentrations of mercury and arsenic were substantially higher in the RBC transfusion blood than in maternal or cord samples, suggesting an additional exposure source, though the extent to which this contributed to the fetal dose remains uncertain.

Our data are not easily comparable with those of previous studies, which differ technically and by design. However, our findings align with those of Kobayashi et al. [77], who used ASQ-3 data from the Japan Environment and Children’s Study (JECS) and reported no association between prenatal mercury exposure in maternal or cord blood and neurodevelopmental delays from 6 months to 4 years—supporting our observation that mercury in cord and maternal blood showed only modest associations with early ASQ-3 scores. Instead, the JECS study identified selenium, not mercury, as associated with a slightly increased risk of developmental delays, highlighting the complexity of nutrient–toxicant interactions. Similarly, arsenic exposure is well known for its potential to cause neurodevelopmental delays in highly contaminated regions; however, findings from low-exposure populations have been mixed [78]. For example, Wang et al. [19] reported that lower arsenic levels in cord blood were associated with poorer neonatal neurobehavior (behavior and passive muscle tone), particularly in infants born to older mothers. Conversely, other longitudinal work has shown that associations at birth may attenuate by 24–36 months [79,80,81]. A closer comparison to our study is the work by Liang et al. [82], who reported that cord arsenic was associated with lower ASQ personal–social scores at 6 months, particularly in girls.

For cadmium, while we did not observe consistent associations with ASQ-3 outcomes, its combined effects with other metals appeared more influential. An unexpected 7.1% increase (95% CI: 0.8–13.8%) in problem-solving scores was associated with combined maternal lead and cadmium levels, though this counterintuitive result likely reflects residual confounding, measurement variability, or chance and should be interpreted cautiously. Studies evaluating cadmium and child development have shown inconsistent findings, with several reporting limited or no evidence of adverse effects [83,84,85,86]. Notably, the extensive JECS cohort analysis by Masumoto et al. [87] reported that prenatal cadmium exposure was associated with developmental delays at 6–18 months, but the association disappeared by 2 years of age—suggesting early but transient effects. In our study, cadmium was the only metal significantly higher in the cord blood of IUBT recipients, though the implications of this elevation in early infancy remain unclear given cadmium’s long half-life (10–30 years) [63].

Although lead is a well-established neurotoxicant even at low levels [88,89,90], we detected no adverse association between cord or maternal lead levels and ASQ-3 domains in our cohort, regardless of IUBT status. This contrasts with recent findings in NICU infants, in whom elevated blood lead levels were associated with lower ASQ-3 scores [91]. Lead levels in our cord samples were approximately four times lower than those reported in NICU populations, which may partly explain these differences. Unexpectedly, we observed a small positive association between lead levels and problem-solving scores in both maternal and cord blood, as well as a non-significant positive trend in fine motor scores associated with lead in RBC transfusions. These paradoxical findings are most likely attributable to unmeasured confounding, small sample size, or developmental variability and should not be interpreted as evidence of benefit. In addition to studies examining maternal and cord blood exposure, few investigations have evaluated metal contamination specifically in transfusion products or its potential implications for fetal or neonatal health. Several previous studies examining medically related metal exposure further contextualize our findings. Falck et al. [92] reported detectable mercury and lead in all donor-packed RBC units used for IUBT, with some transfusions delivering fetal doses far exceeding estimated intravenous reference doses, demonstrating that transfusion-mediated exposure can substantially contribute to fetal metal burden. Similarly, Al-Saleh et al. [91] observed that preterm neonates in the NICU received clinically significant amounts of mercury, lead, cadmium, arsenic, and manganese via total parenteral nutrition and RBC transfusions, with higher neonatal metal levels associated with lower ASQ-3 domain scores at 2 months. Additional evidence from Celik et al. [93] showed transplacental transfer of multiple metals and ongoing neonatal exposure in the NICU, supporting the concept of cumulative perinatal metal burden across multiple biomatrices. Moreover, Aly et al. [94] found that 19.2% and 5.9% of neonatal blood products exceeded recommended concentrations for lead and cadmium, respectively, with statistically significant concurrent exposure to lead, mercury, and cadmium. Taken together, these studies indicate that blood products and NICU-related interventions may represent important, though understudied, contributors to perinatal metal exposure—consistent with our observations of detectable metal levels in IUBT transfusion units. However, because prior studies did not specifically evaluate neurodevelopment following IUBT, and given the modest and imprecise associations observed in our cohort, the implications of transfusion-related metal exposure for early developmental outcomes remain uncertain.

Overall, the observed associations were modest in magnitude, imprecise in several cases, and limited by the early timing of ASQ-3 assessment, which is designed for screening rather than diagnosis. These results should therefore be viewed as exploratory and hypothesis-generating rather than definitive.

### 4.5. Study Limitations and Strengths

This study has several limitations that warrant acknowledgment. First, the relatively small sample size may have limited the statistical power to detect weaker associations between prenatal metal exposure levels and neurodevelopmental outcomes. This constraint was particularly relevant for the subgroup with complete ASQ-3 data and for infants who underwent IUBT, increasing the potential for type II error. Second, although the models were adjusted for several maternal and perinatal characteristics, additional factors that could influence both metal exposure and neurodevelopmental scores were not fully accounted for. These include parity; the infant’s sex, given the known sex-specific sensitivity to neurotoxic metals; and maternal lifestyle factors such as passive smoking and dietary intake of mercury- or arsenic-containing foods (e.g., seafood). Furthermore, important determinants of susceptibility—such as genetic factors, socioeconomic status, and maternal nutritional status—were not incorporated into the multivariable models because the available data were limited, incomplete, or insufficient given the modest sample size. These variables are known to influence both toxicant exposure pathways and neurodevelopmental vulnerability [95,96,97]. Third, the absence of cord blood samples not collected by nurses in the delivery room for the study presents another limitation. Fourth, assessing infants at 2 months may be too early to reliably estimate neurodevelopmental outcomes. Fifth, the ASQ-3, while being more sensitive and specific in older infants or children [98], is a parental questionnaire-based assessment. This method may introduce parental bias, as reporting can be influenced by factors such as health literacy, maternal stress, and parental perception [99,100,101]. Discrepancies between parent-reported measures and professionally administered assessments have also been documented, particularly in very young infants [98]. Furthermore, the ASQ-3 administered at 2 months serves as an early screening tool rather than a diagnostic assessment, and its sensitivity for detecting subtle neurodevelopmental delays is limited at this age; predictive validity improves after 6 months [102]. Sixth, some infant anthropometric data (weight and length) were incomplete or imprecise due to phone-based data collection at follow-up. Seventh, residual confounding from unmeasured factors may have contributed to unexpected associations. Eighth, simultaneous exposure to other environmental pollutants—including broader chemical mixtures not captured in this study—was not assessed. Evidence from exposome-based research shows that multi-pollutant mixtures can modify or obscure associations between individual toxicants and neurodevelopment [103], suggesting that unmeasured co-exposures may have influenced our findings. Ninth, not all RBC transfusions were analyzed for metal content because nursing staff failed to collect the residual samples. Tenth, the exploratory nature of the study and the high intercorrelation among metal exposures meant that no formal correction for multiple comparisons was applied; strict methods such as Bonferroni or FDR may substantially inflate type II error in studies of correlated exposures. Therefore, results were interpreted with emphasis on effect sizes, confidence intervals, and consistency across matrices rather than *p*-values alone [47,48]. Eleventh, potential post-analytical contamination of RBC units cannot be completely excluded. Although samples were handled according to established trace-metal protocols, contamination during blood bank processing, storage, or sampling could influence measured concentrations and should be considered when interpreting transfusion-related exposure. Twelfth, because sampling adequacy for PCA was low (KMO 0.39–0.52), the extracted patterns should be interpreted as exploratory rather than as definitive latent components. Low KMO values and small sample sizes are known to compromise the stability, interpretability, and reproducibility of PCA solutions, and PCA under such conditions is recommended only for preliminary pattern detection rather than formal component identification [104]. Lastly, the study measured total arsenic in all matrices, limiting the ability to distinguish between organic and inorganic forms, which differ substantially in toxicity.

Despite these limitations, the study has several significant strengths, including its prospective design and the use of multiple matrices for exposure assessments at two critical points: the third trimester and at delivery. The integration of maternal, cord, and transfusion samples provides a more comprehensive characterization of fetal metal exposure than studies relying on a single biological matrix. Additionally, the inclusion of IUBT-exposed infants offers rare insight into a clinically unique high-risk population.

### 4.6. Future Directions

Future research should incorporate long-term neurodevelopmental follow-up beyond early infancy—such as at 12, 18, and 24 months—when ASQ-3 and other developmental tools have more substantial predictive value. Larger multicenter studies are needed to enhance generalizability and to allow robust adjustment for genetic, socioeconomic, dietary, and environmental covariates. Additionally, efforts to prevent metal contamination in transfusion products should include systematic screening, improved blood bank handling procedures, and targeted quality control measures. Speciation of arsenic and expanded analysis of other toxicants would further clarify exposure pathways and biological mechanisms.

## 5. Conclusions

This prospective study examined prenatal exposure to mercury, cadmium, lead, and arsenic among pregnant women, including those receiving IUBT, and explored their infants’ early neurodevelopment at 2 months of age. Heavy metals were detected in a substantial proportion of maternal and cord blood samples, as well as in RBC transfusion units used for IUBT, indicating that multiple pathways of perinatal exposure may exist. Although most concentrations were within internationally accepted reference ranges, the presence of neurotoxic metals in all three matrices remains noteworthy from a public health perspective.

Using the ASQ-3 as an early screening tool, we observed modest and imprecise associations between selected metal exposure levels and reductions in specific developmental domain scores. These associations became slightly stronger numerically after adjustment for IUBT, and metal levels in RBC transfusions showed additional suggestive relationships with gross motor scores. However, the magnitude of these changes was small (<6%), confidence intervals were wide, and findings did not withstand more stringent statistical correction. Accordingly, these preliminary results should be interpreted with caution and viewed as hypothesis-generating rather than indicative of clinically meaningful developmental effects.

Overall, the study highlights the potential for transfusion products to contribute to fetal metal exposure. Still the evidence does not establish IUBT as a definitive or independent source of neurodevelopmental risk. Our findings instead point to the importance of ongoing surveillance of metal content in maternal–fetal biomatrices and maintaining stringent quality control of medical products used during pregnancy. Future research with larger cohorts and longer neurodevelopmental follow-up is needed to clarify the relevance of these early-life exposures and to determine whether the suggestive associations observed here persist or evolve later in infancy and childhood.

## Figures and Tables

**Table 1:** Summary of blood/RBC sample preparation, instrumentation, calibration, accuracy, and precision procedures.

Category	Details
Sample Preparation	50 µL whole blood or RBC sample diluted 1:50 Diluent: 0.5% nitric acid, 0.05% Triton-X, 2% methanol (*v*/*v*) Additives: 250 µg/L gold; 0.1 µg/L internal standards [rubidium-103 for arsenic; and iridium-193 for lead, mercury, cadmium) Trace-metal-free materials used throughout
Instrumentation	Inductively coupled plasma–mass spectrometry (ICP-MS; Perkin Elmer NexION^®^ 2000) Diluent signal subtracted from calibrators, quality control, and samples
Calibration	Calibration range for all analytes: 0.25–4.0 µg/L Linearity, expressed as the correlation coefficient (mean ± SD), was as follows: cadmium 0.9997 ± 0.0003 (*n* = 11); lead 0.9994 ± 0.0006 (*n* = 11); arsenic 0.9998 ± 0.0003 (*n* = 10); mercury 0.9998 ± 0.0002 (*n* = 10)
Analytical Accuracy	Verified using external reference materials and internal quality control samples
Between-Run Precision	Pooled blood spiked at 0.75, 1.5, 3 µg/L: Recovery (%): cadmium 100–100.4; lead 99.9–101.3; arsenic 101.4–104.3; mercury 97.7–101.9 Relative standard deviation (RSD (%): <5% except lead (6.5%) and mercury (7.5%) at 0.75 µg/L
Within-Run Precision	Ten replicates at 0.3, 0.75, 1.5 µg/L: RSD (%): cadmium 2.0–3.4; lead 2.6–4.6; arsenic 2.4–4.1; mercury 2.3–3.7
Standard Reference Materials	* ClinChek^®^ (three levels): Cadmium: 1.93, 4.05, 7.22 µg/L Lead: 34.68, 87.3, 239.4 µg/L Arsenic: 2.83, 9.33, 18.15 µg/L Mercury: 3.57, 7.81, 17.53 µg/L All values within certified ranges
** Limit of Detection (LOD)	Cadmium: 0.002 µg/L Lead: 0.044 µg/L Arsenic: 0.001 µg/L Mercury: 0.002 µg/L
*** Limit of Quantitation (LOQ)	Cadmium: 0.006 µg/L Lead: 0.132 µg/L Arsenic: 0.004 µg/L Mercury: 0.007 µg/L

* ClinChek^®^, Recipe Chemicals and Instruments GmbH, Munich, Germany); ** LOD was calculated as the standard deviation (SD) of ten blank replicates multiplied by Student’s t value for *n*−1 degrees of freedom at the 99% confidence level. *** LOQ was calculated as 10 × SD of the same blank replicates.

**Table 2:** (a) Maternal and infant characteristics and neurodevelopmental outcomes by intrauterine blood transfusion (IUBT) status. Continuous variables are summarized as mean, median, minimum, and maximum values. Categorical variables are expressed as frequencies and percentages. Group differences for continuous variables were assessed using the Mann–Whitney U test, and categorical variables were compared using the chi-squared (χ^2^) test. Infant ASQ-3 domain scores were age-corrected for prematurity before scoring. (b) Maternal, fetal, and transfusion-related metal exposure and infant neurodevelopmental outcomes by IUBT status. Maternal (third-trimester) and cord blood metal concentrations (µg/L) are summarized for women who did and did not receive intra-uterine blood transfusion (IUBT). Neurodevelopment at 2 months was assessed using ASQ-3 domain scores. Continuous variables are presented as mean, median, minimum, and maximum values. Group comparisons were performed using the Mann–Whitney U test.

a											
Continuous Variables: Demographic/Clinical/ASQ-3-Domains	No IUBT Recipients					IUBT Recipients					*p*-Value
	*N*	Mean	Median	Min	Max	*N*	Mean	Median	Min	Max	
Mother’s age (years)	60	32.8	31.7	25.8	45.7	30	35.8	36.1	29.6	46.0	0.001
Weight (Kg)	60	79.6	78.5	47.9	139.0	30	80.4	78.5	44.3	126.0	0.891
Height (cm)	60	158.6	157.0	144.5	177.0	30	160.2	160.0	150.0	174.0	0.217
BMI (Kg/m^2^)	60	31.6	31.2	21.6	49.8	30	31.1	30.2	19.7	48.0	0.626
Number of gestational weeks	60	33.4	34.0	27.0	39.0	30	25.3	25.5	17.0	32.0	0.000
Number of live births	42	2.1	2.0	1.0	6.0	30	2.9	3.0	1.0	6.0	0.008
Number of stillbirths	6	1.5	1.5	1.0	2.0	20	1.6	1.0	1.0	5.0	0.752
Miscarriages	29	1.7	1.0	1.0	7.0	17	2.2	2.0	1.0	9.0	0.065
Live births less than 38 weeks of gestational age	5	1.2	1.0	1.0	2.0	12	1.6	1.5	1.0	3.0	0.249
Number of amalgam fillings	41	3.6	4.0	1.0	12.0	20	3.7	4.0	1.0	6.0	0.224
Gestational age (weeks) at delivery	60	38.3	38.0	34.0	41.0	30	34.2	35.0	29.0	38.0	0.000
Newborn’s Weight (Kg)	55	3.0	3.0	2.3	4.3	23	2.4	2.5	1.5	3.0	0.000
Newborn’s height (cm)	41	50.0	51.0	44.0	57.0	12	45.2	45.5	38.0	50.0	0.003
Head circumference (cm)	22	35.7	34.5	32.0	47.0	10	32.8	32.3	30.0	36.5	0.000
Crown–heel length (cm)	39	49.9	50.0	44.0	57.0	12	45.2	45.5	38.0	50.0	0.000
Apgar 1-min score	49	7.9	8.0	5.0	9.0	20	5.9	6.5	0.0	9.0	0.001
Apgar 5-min score	49	8.8	9.0	7.0	10.0	20	7.6	8.0	5.0	10.0	0.001
Infant’s body weight (kg) at 2 months	47	5.6	5.5	4.0	8.0	18	8.4	6.0	4.3	50.0	0.139
Infant’s height (cm) at 2 months	36	59.5	58.5	49.0	75.5	13	58.3	60.0	50.0	66.0	0.592
ASQ-3-Communication	55	49.18	50.0	20.0	60.0	19	50.53	50.0	30.0	60.0	0.575
ASQ-3-Gross motor	54	51.30	52.5	25.0	60.0	19	51.84	60.0	30.0	60.0	0.527
ASQ-3-Fine motor	55	51.64	55.0	30.0	60.0	19	47.37	50.0	30.0	60.0	0.229
ASQ-3-Problem-solving	55	54.55	60.0	20.0	60.0	19	52.37	55.0	20.0	60.0	0.131
ASQ-3-Personal–social	55	50.00	50.0	30.0	60.0	19	49.21	50.0	30.0	60.0	0.964
Categorical variables	Non-IUBT recipients *N* (%)					IUBT recipients, *N* (%)					*p*-value
Educational level (≤12/>12 years schooling)	6 (6.7%)/54 (60%)					9 (10%)/21 (23.3%)					0.032
Work status (Current/Past and/or Never)	28 (31.1%)/32 (35.6%)					7 (7.8%)/23 (25.6%)					0.042
Family income in SR (≤10,000/>10,000/Unknown or refused)	17 (18.9%)/27 (30%)/16 (17.8%)					6 (6.7%)/12 (13.3%)/12 (13.3%)					0.406
Current pregnancy conceived through in vitro fertilization (Yes/No)	6 (6.7%)/54 (60%)					0 (0%)/30 (33.3%)					0.08
Have any children from previous pregnancies experienced neurological problems? (Yes/No)	1 (1.1%)/58 (65.2%)					3 (3.4%)/27 (30.3%)					0.109
Health status (Diseased/None)	35 (38.9%)/25 (27.8%)					18 (20%)/12 (13.3%)					0.532
Smoking cigarettes and/or water pipes (Yes/No)	2 (2.2%)/58 (64.4%)					1 (1.1%)/29 (32.2%)					0.709
Living with a smoker, including spouse or family member (Yes/No)	15 (16.7%)/45 (50%)					13 (14.4%)/17 (18.9%)					0.077
Living in a polluted area (Yes/No)	12 (13.3%)/48 (53.3%)					9 (10%)/21 (23.3%)					0.290
Dental amalgam (Yes/No)	43 (47.8%)/17 (18.9%)					20 (22.2%)/10 (11.1%)					0.626
Seafood intake (Yes/No)	51 (56.7%)/9 (10%)					125 (27.8%)/5 (5.6%)					0.837
Seafood frequency (Weekly or monthly/Seldom)	38 (50%)/13 (17.1%)					19 (25%)/6 (7.9%)					0.888
Newborn gender (Female/Male)	23 (20.1%)/34 (40%)					15 (17.6%)/13 (15.3%)					0.249
b											
Maternal Exposure	No IUBT Recipients					IUBT Recipients					*p*-Value
	*N*	Mean	Median	Min	Max	*N*	Mean	Median	Min	Max	
Mercury levels in the blood—third trimester (µg/L)	60	0.48	0.36	0.00	2.15	29	0.41	0.34	0.00	1.07	0.993
Cadmium levels in blood—third trimester (µg/L)	60	0.55	0.18	0.01	4.82	29	0.82	0.26	0.00	3.53	0.386
Lead levels in blood—third trimester (µg/L)	60	6.04	3.94	0.02	82.42	29	5.03	4.26	0.72	19.57	0.766
Arsenic levels in blood—third trimester (µg/L)	60	0.40	0.35	0.00	1.70	29	0.42	0.24	0.00	1.86	0.944
Fetal exposure	No IUBT recipients					IUBT recipients					*p*-value
	*N*	Mean	Median	Min	Max	*N*	Mean	Median	Min	Max	
Mercury levels in cord blood (µg/L)	40	1.01	0.85	0.00	4.72	21	0.68	0.52	0.05	2.03	0.129
Cadmium levels in cord blood (µg/L)	40	0.03	0.02	0.00	0.14	21	0.35	0.08	0.00	2.37	0.003
Lead levels in cord blood (µg/L)	40	3.88	3.21	1.55	9.29	21	4.27	4.35	0.23	12.05	0.820
Arsenic levels in cord blood (µg/L)	40	0.19	0.07	0.00	2.54	21	0.38	0.09	0.00	3.56	0.132
Blood transfusion exposure	No IUBT recipients					IUBT recipients					*p*-value
	*N*	Mean	Median	Min	Max	*N* *	Mean	Median	Min	Max	
Mercury in the blood transfusion (µg/L)						29	1.516	0.746	0.001	28.225	NA
Cadmium in blood transfusion (µg/L)						29	0.924	0.749	0.001	3.947	NA
Lead in blood transfusion (µg/L)						29	14.690	12.798	3.116	41.439	NA
Arsenic in blood transfusion (µg/L)						29	0.746	0.436	0.0007	8.423	Na

* Transfusion-related exposure reflects metal concentrations measured in residual samples from 124 packed RBC transfusion units administered to 29 IUBT recipients.

**Table 3:** Regression models estimating the associations between metal concentrations in maternal blood, cord blood, RBC transfusion samples, and combinations of metals (via PCA components) and the five ASQ-3 developmental domains. Results are presented as regression coefficients (β) with 95% confidence intervals (CI). Bolded values indicate statistically significant associations (* *p* < 0.01; ** *p* < 0.01) or borderline associations (*** *p* < 0.1).

Matrix	Models	Communication			Gross Motor			Fine Motor			Problem-Solving			Personal–Social		
		β	95% CI		β	95% CI		β	95% CI		β	95% CI		β	95% CI	
Maternal	Mercury	0.001	−0.030	0.033	−0.005	−0.034	0.024	0.019	−0.01	0.047	−0.002	−0.035	0.031	−0.031 **	**−0.056**	**−0.007**
	Cadmium	−0.006	−0.047	0.036	−0.005	−0.043	0.032	0.022	−0.016	0.06	−0.039 ***	**−0.082**	**0.004**	−0.014	−0.048	0.019
	Lead	0.007	−0.044	0.058	−0.011	−0.058	0.035	−0.014	−0.061	0.033	0.045 ***	**−0.008**	**0.097**	0.019	−0.023	0.06
	Arsenic	0.007	−0.036	0.05	0.009	−0.03	0.048	0.017	−0.023	0.056	−0.017	−0.063	0.028	−0.015	−0.05	0.02
	PC1 (mercury and arsenic)	0.007	−0.050	0.063	0.005	−0.047	0.056	0.031	−0.021	0.083	−0.018	−0.078	0.042	−0.045 ***	**−0.090**	**0.001**
	PC2 (lead & cadmium)	0.008	−0.052	0.067	−0.01	−0.064	0.044	−0.022	−0.077	0.033	0.069 **	**0.008**	**0.129**	0.011	−0.037	0.060
Cord blood	Mercury	−0.018	−0.071	0.035	−0.011	−0.06	0.038	0.004	−0.051	0.06	−0.049	−0.113	0.016	−0.028	−0.073	0.016
	Cadmium	−0.024	−0.065	0.017	−0.019	−0.057	0.019	−0.002	−0.045	0.042	0.01	−0.043	0.062	−0.007	−0.042	0.029
	Lead	−0.036	−0.156	0.083	0.051	−0.058	0.161	0.107 ***	**−0.012**	**0.227**	0.047	−0.102	0.196	0.03	−0.072	0.132
	Arsenic	0.005	−0.02	0.029	−0.006	−0.028	0.017	−0.003	−0.028	0.023	−0.04 *	**−0.068**	**−0.012**	0.002	−0.019	0.023
	PC1 (mercury, cadmium & arsenic)	0.006	−0.065	0.076	−0.004	−0.069	0.061	0.002	−0.071	0.074	−0.089 **	**−0.172**	**−0.006**	−0.012	−0.072	0.048
	PC2 (lead)	−0.027	−0.105	0.051	0.036	−0.036	0.107	0.071 ***	**0.072**	**−0.007**	0.041	−0.056	0.138	0.014	−0.053	0.081
Maternal and cord	PC1 (arsenic in maternal, mercury arsenic & cadmium in cord)	0	−0.070	0.071	0	−0.065	0.064	0.013	−0.060	0.085	−0.077 ***	−0.161	0.007	−0.021	−0.081	0.039
	PC2 (mercury, lead & cadmium in maternal)	−0.022	−0.099	0.056	0.001	−0.070	0.073	0.021	−0.059	0.101	0.037	−0.058	0.133	−0.032	−0.097	0.033
RBC transfusion	^a^ Mercury	−0.001	−0.144	0.142	−0.116 **	**−0.220**	**−0.012**	−0.094	−0.249	0.061	−0.087 ***	**−0.186**	**0.012**	−0.014	−0.179	0.151
	^a^ Cadmium	0.012	−0.061	0.085	−0.021	−0.087	0.046	0.016	−0.069	0.102	−0.018	−0.077	0.040	0.014	−0.070	0.098
	^a^ Lead	0.338	−0.199	0.875	−0.006	−0.549	0.537	0.530 ***	**−0.052**	**1.112**	0.254	−0.189	0.698	0.032	−0.647	0.711
	^a^ Arsenic	0.008	−0.055	0.071	−0.045 ***	**−0.094**	**0.005**	−0.03	−0.101	0.042	−0.02	−0.070	0.029	0.014	−0.059	0.087
	^a^ PC1 (mercury & arsenic)	0.021	−0.155	0.196	−0.138 **	**−0.269**	**−0.007**	−0.096	−0.292	0.101	−0.068	−0.204	0.067	0.012	−0.192	0.216
	^a^ PC2 (lead & cadmium)	0.117	−0.109	0.344	−0.072	−0.289	0.145	0.179	−0.075	0.433	−0.01	−0.206	0.186	0.058	−0.218	0.333
All matrices	PC1 (mercury & arsenic)	0.063	−0.296	0.421	−0.18	−0.568	0.209	0.076	−0.428	0.579	0.037	−0.287	0.361	0.002	−0.475	0.479
	PC2 (lead & cadmium)	−0.083	−0.440	0.274	−0.163	−0.574	0.248	−0.093	−0.601	0.414	−0.104	−0.406	0.197	−0.023	−0.507	0.462

Models adjusted for mother’s age, BMI, gestational age at delivery, family income, and health status. ^a^ Models adjusted for mother’s age, BMI, number of RBC units received, gestational age at delivery, family income, and health status.

**Table 4:** Regression models separately estimating the associations between metal concentrations in maternal blood, cord blood, RBC transfusion samples, and combinations of metals (via PCA components) and the five ASQ-3 developmental domains, adjusted for IUBT status. Results are presented as regression coefficients (β) with 95% confidence intervals (CI). Bolded values indicate statistically significant associations (* *p* < 0.01; ** *p* < 0.01) or borderline associations (*** *p* < 0.1).

	Models	Communication			Gross Motor			Fine Motor			Problem-Solving			Personal–Social		
		β	95% CI		β	95% CI		β	95% CI		β	95% CI		β	95% CI	
Maternal blood	Mercury	0.003	−0.029	0.035	−0.008	−0.037	0.021	0.015	−0.014	0.044	−0.003	−0.037	0.031	−0.035 **	**−0.06**	**−0.01**
	Cadmium	−0.005	−0.047	0.036	−0.005	−0.043	0.032	0.022	−0.016	0.059	−0.039 ***	**−0.082**	**0.004**	−0.014	−0.048	0.019
	Lead	0.006	−0.046	0.057	−0.009	−0.055	0.038	−0.01	−0.057	0.036	0.046 ***	**−0.007**	**0.1**	0.021	−0.02	0.063
	Arsenic	0.011	−0.034	0.055	0.003	−0.037	0.043	0.01	−0.03	0.05	−0.021	−0.068	0.025	−0.022	−0.058	0.014
	PC1 (mercury & arsenic)	0.011	−0.048	0.070	−0.004	−0.057	0.049	0.024	−0.031	0.076	−0.024	−0.086	0.039	−0.055 **	**−0.101**	**−0.009**
	PC2 (lead & cadmium)	0.007	−0.053	0.067	−0.008	−0.062	0.046	−0.019	−0.073	0.035	0.07 **	**0.009**	**0.131**	0.013	−0.035	0.062
Cord blood	Mercury	−0.012	−0.066	0.042	−0.018	−0.068	0.031	−0.007	−0.061	0.047	−0.063 **	**−0.125**	**0**	−0.03	−0.076	0.016
	Cadmium	−0.037 ***	**−0.079**	**0.006**	−0.012	−0.052	0.029	0.014	−0.03	0.058	0.028	−0.025	0.081	−0.006	−0.045	0.032
	Lead	−0.021	−0.143	0.100	0.037	−0.074	0.148	0.085	−0.033	0.204	0.02	−0.128	0.168	0.03	−0.076	0.135
	Arsenic	0.006	−0.019	0.03	−0.006	−0.029	0.016	−0.004	−0.028	0.02	−0.041 *	**−0.068**	**−0.015**	0.002	−0.019	0.023
	PC1 (mercury, cadmium & arsenic)	0.017	−0.055	0.089	−0.015	−0.081	0.050	−0.016	−0.087	0.056	−0.112 *	**−0.192**	**−0.033**	−0.014	−0.076	0.049
	PC2 (lead)	−0.016	−0.096	0.064	0.025	−0.048	0.098	0.055	−0.023	0.134	0.021	−0.076	0.119	0.013	−0.057	0.083
Maternal and cord	PC1 (arsenic in maternal, mercury in cord, arsenic & cadmium)	0.011	−0.060	0.083	−0.012	−0.077	0.054	−0.004	−0.076	0.067	−0.101 **	**−0.182**	**−0.019**	−0.023	−0.085	0.039
	PC2 (mercury, lead & cadmium in maternal)	−0.013	−0.091	0.066	−0.009	−0.081	0.063	0.006	−0.072	0.085	0.022	−0.073	0.117	−0.034	−0.101	0.033

Models adjusted for mother’s age, BMI, receipt of IUBT, gestational age at delivery, family income, and health status.

## Data Availability

The data supporting the findings of this study are available from the corresponding author upon reasonable request. Access to the data is restricted due to institutional and ethical requirements to protect participant privacy.
